# Imaging of complicated frontal sinusitis

**DOI:** 10.11604/pamj.2017.26.209.11817

**Published:** 2017-04-19

**Authors:** Naourez Kolsi, Ahmed Zrig, Hamdi Chouchène, Rachida Bouatay, Khaled Harrathi, Jamel Koubaa

**Affiliations:** 1 Department of Otorhinolaryngology, Head and Neck Surgery at “Fattouma Bourguiba” Hospital, Monastir, Tunisia; 2 Department of Radiology at “Fattouma Bourguiba” Hospital, Monastir, Tunisia

**Keywords:** Frontal sinusitis, computed tomography, magnetic resonance imaging, complications

## Abstract

Complications occur in 3% of the cases of frontal sinusitis. These are mainly oculo-orbital, intracranial and osteomyelitis. Our aim is to describe the contribution of different imaging modalities in the diagnosis of these complications and their post-treatment monitoring. within a 15 years period (2000-2014), 10 patients with complicated frontal sinusitis were included into this retrospective study. 10 patients (9 males) aged 9 to 70 year old (mean 28). Two of these patients (20%) had a history of craniofacial trauma. Frontal headache was present in all cases (100%), frontal swelling in 8 cases (80%) and unilateral palpebral edema in 3 cases (30%). A CT scan of the face and brain was performed in all cases and revealed frontal osteomyelitis in 6 cases (60%), extradural empyema in 3 cases (33%), intracranial frontal abscess in 2 cases (20%) and occulo-orbital complications in 3 cases (30%). Magnetic resonance imaging was performed in one patient and demonstrated thrombosis of the upper longitudinal sinus. 40% of our patients associated 2 complications. Cross-sectional imaging is important in early and accurate diagnosis of complicated frontal sinusitis.

## Introduction

Face Sinus infections or sinusitis are frequent community infections, which evolve favorably in the great majority of cases. The occurrence of complications in acute sinusitis has declined markedly since the advent of antibiotics. However, certain forms of sinusitis are known to be more prone to suppurative complications, in particular frontal sinusitis which owes their severity to their potential life-threatening intracranial extension. This is explained by the intimacy of the anatomical relationships of the frontal sinus with the anterior cerebral fossa and with the orbit. Therefore, early diagnosis and urgent initiation of appropriate treatment are the only guarantees to reduce the morbidity of complicated frontal sinusitis. Cutting imaging plays a pivotal role in these complications early and accurate diagnosis.

## Methods

**Type of study**: our study is a descriptive retrospective study of ten patients hospitalized in our department, for complicated frontal sinusitis, either singly or in the context of pan-sinusitis, over a 15-year period from 2000 to 2014. All our patients had a complete ENT examination with a nasal endoscopy, a neurological examination, an ophthalmological examination with a request for a complete biological balance including renal function, an inflammatory balance and an operability and pre-anesthetic report. All our patients underwent a Computed Tomography (CT) scan of the facial and cerebral mass with intravenous injection of contrast medium, performed either in thin and close axial sections (every 2 to 3 mm), for examinations performed on or in volume acquisition with infra-millimetric cuts on a multicore scanner and a double fenestration study (bone and soft tissue) in multiplanar reconstructions. The Computed Tomography allowed us to analyze the osseous walls of the frontal sinus, the state of the cerebral parenchyma and the orbital content and the vascular permeability. A Magnetic Resonance Imaging (MRI) was performed in a single patient (who had an extradural empyema observed at CT) in front of the persistence of headache despite antibiotic therapy, in search of pre-suppurative encephalitis. The protocol consisted of a brain and facial mass exploration with FLAIR, diffusion and volume acquisition sequences with injection of Gadolinium. The study of the facial mass was carried out by series of T2, T1 and T1Fat Sat weights with IV injection of Gadolinium.

**Collection of data**: the data were collected on the basis of an analytical grid of medical records with analysis of the following elements: age and gender, antecedents, functional signs, clinical examination data, biological examination data, radiological examination data, therapeutic modalities, post-therapeutic evolution.

## Results

**Figure 1:**
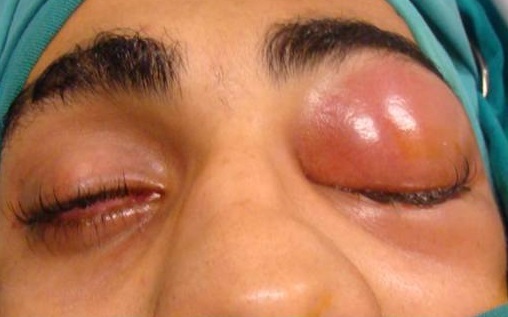
Left upper palpebral abscess with lower palpebral edema complicating frontal homolateral sinusitis

Our study series included 10 patients of middle age 28 years with extremes of 9 and 70 years. A male predominance was observed (sex ratio = 9). Two of our patients had a history of craniofacial trauma and two others had chronic sinusitis. The clinical symptomatology was dominated by headaches reported by all patients (100%), associated with the presence of a frontal swelling in 8 cases (80%) purulent rhinorrhea in 4 cases (40%), Nasal obstruction in 8 cases (80%) and periorbital swelling in 2 cases (20%). A fever estimated at 39 ° was found in 4 cases (40%). The physical examination found inflammatory frontal swelling in 8 cases (80%), diffuse palpebral edema in 5 cases (50%), periorbital swelling in 2 cases (20%). The nasal endoscopy showed the presence of pus in the middle meatus in 7 cases (70%). The Ophthalmological examination found non-axillary exophthalmia non-reducible in 2 cases, upper palpebral enlargement in two cases Figure 1 and diffuse palpebral edema in 5 cases (50%). The neurological examination revealed neither sensory-motor deficit nor meningeal signs.

**Figure 2:**
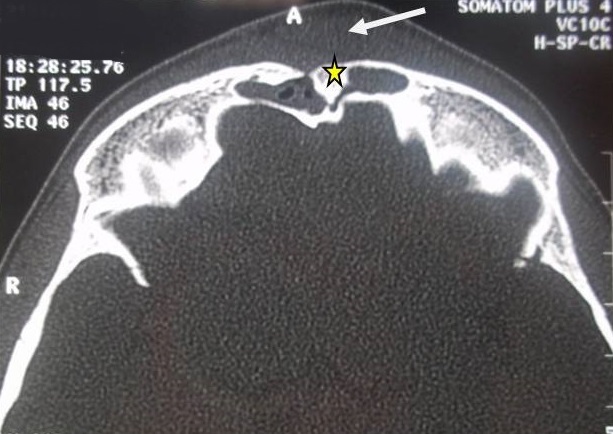
Brain CT in axial section, bone window showing a post-traumatic bone embedment of the external table of frontal sinus (star) filling and marked thickening of the soft opposite parts (arrow)

**Figure 3:**
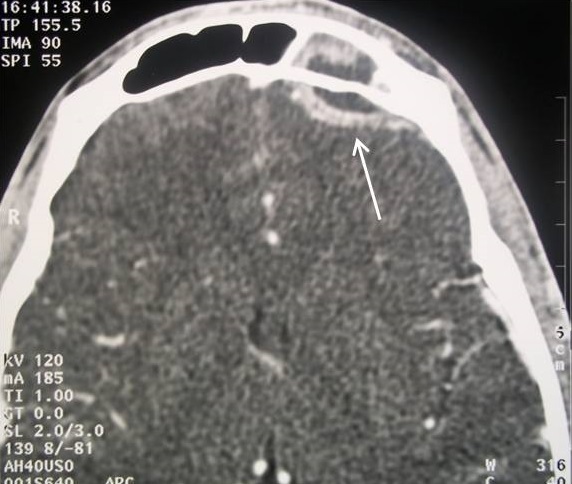
CT in axial cuts after PDC injection in parenchymatous window showing a hypodensic collection on both sides of the internal table of the left frontal sinus with peripheral contrast (arrow) in favor of a left frontal extradural empyema complicating sinusitis Frontal homolateral

**Figure 4:**
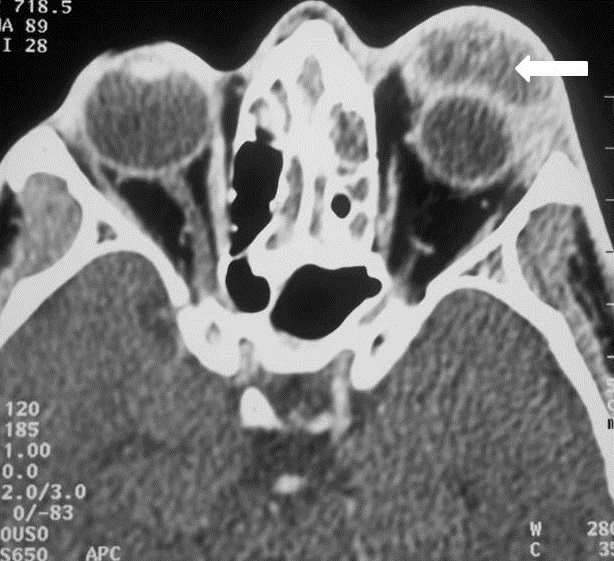
CT of the facial mass (from the same patient in figure 1) in axial sections after PDC injection in the soft-tissue window showing a presptal hypodense orbital image with peripheral contrast check (arrow): left prespetal abscess (Chandler’s Stage I) complicating frontal homolateral sinusitis

**Imaging**: the CT of the facial and cerebral mass with injection of PDC showed a frontal sinus filling in favor of frontal sinusitis in all these patients. Preseptal Cellulitis was noted in 2 cases. A frontal osteomyelitis was objectified in 60% of our patients, with thickening of the opposite soft parts. The CT also detected a fracture of the external table of the frontal sinus in a patient, which caused an exclusion of the sinus by a bone callus and which seems to be the factor favoring the complication of the frontal sinusitis in this patient Figure 2. Intracranial complications such as extradural Figure 3 or subdural empyema, cerebral abscess, thrombosis of the upper longitudinal sinus were noted in 50% of the patients and the oculo-orbital affections: Preseptal Cellulitis Figure 4 and periosteal abscesses were found in 30% Table 1.

**Table 1:** CT data according to the different types of complications of frontal sinusitis observed in our patients

CT of the facial and cerebral mass		Patients Nb	
Frontal osteomyelitis		6	
Intracranial complications	Extradural empyema (Figure 3)	3	5
	Frontal abscess	2	
Oculo-orbit Complications	preseptal Cellulitis (Stage I of Chandler) (figure 4)	2	3
	Subperiosteal abscess (Stage III of Chandler)	1	

**Figure 5:**
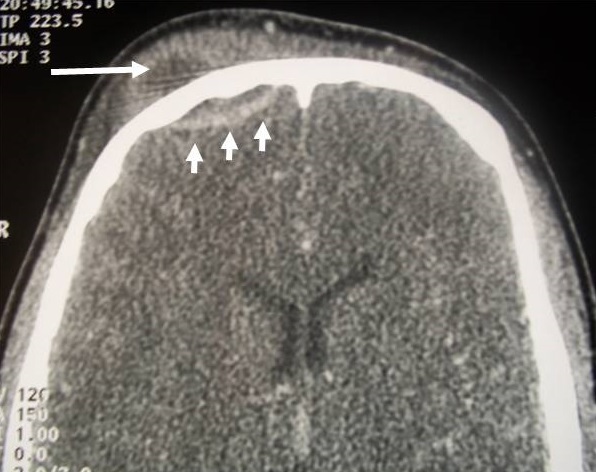
Subperiosteal abscess of the right anterior frontal area (long arrow) associated with a homolateral extradural empyema (short arrows) complicating right frontal sinusitis: a typical aspect of "Pott’s Puffy tumour"

**Figure 6:**
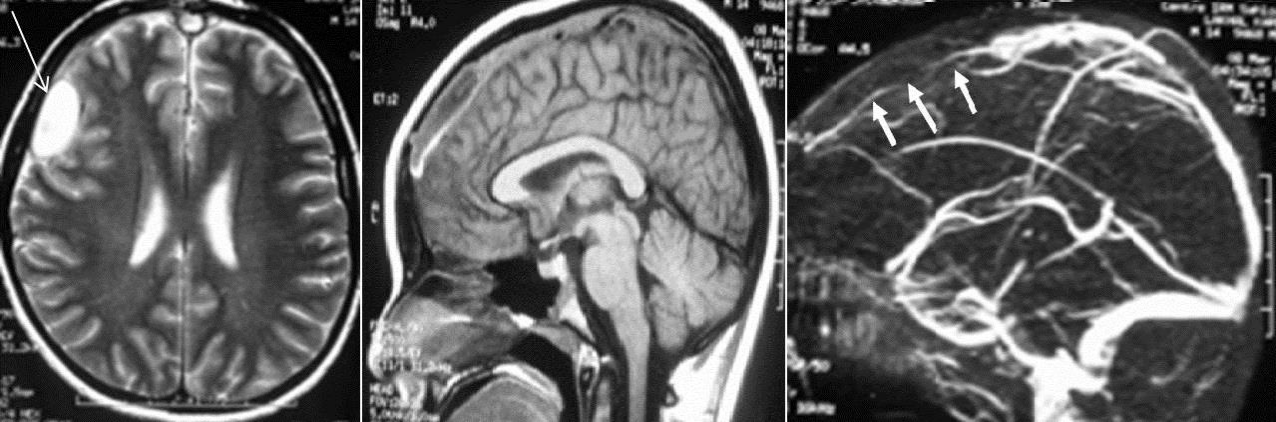
MRI and cerebral venous MRI showing a right frontal extradural empyema (long arrow) associated with a thrombophlebitis of the upper longitudinal sinus complicating frontal sinusitis (short arrows)

**Injury associations**: it was noted that 40% of our patients had two types of associated complications. Two patients had frontal sinusitis complicated with a frontal osteomyelitis associated with a subperiosteal frontal abscess, thus integrating into a particular pathological entity called "Pott's Puffy tumor". One of these patients had also an associated extradural empyema **Figure 5**. The MRI of the facial and cerebral mass was performed in a single patient and showed a right frontal extradural empyema associated with a thrombophlebitis of the upper longitudinal sinus **Figure 6**.

**Therapeutic aspects**: our patients received a triple broad spectrum intravenous antibiotic therapy based mainly on the combination of cefotaxime, metronidazole and fosfomycin or vancomycin for an average duration of 3 weeks followed by an oral relay for an average total duration of 2 months. Surgical drainage was performed in 8 cases: 4 external and 4 combined (endonasal + external). Imaging was with a great benefit in the monitoring of these patients and a CT scan of the facial and cerebral control was systematically requested in 80% of cases after an average of 45 days. The follow up developments were favorable in all cases.

## Discussion

The orbital and endocranial complications of acute bacterial sinusitis present a diagnostic and therapeutic problem. The sinusites of the base of the skull in particular the acute frontal sinusitis are the most providers of this type of complications. The frontal sinus is considered to be the sinus most involved in the endocranial complications either in isolation or as part of a pan sinusitis [1-4]. El Hakim and al [5] Study showed that the prevalence of intracranial complications of acute sinusitis in the pediatric population was significantly higher (30.4%) in children with frontal sinusitis than in those without frontal sinus involvement (2.12%). In this study [5], the authors insist that special attention should be given to children with acute frontal sinusitis in whom the risk of morbid complications is increased. In the literature, it is well noted by the great majority of authors that the risk of complications in acute sinusitis is related to age (children and adolescents) [1, 6, 7], to the field (diabetes, immune-suppression) and to anatomical factors favoring that they are congenital or acquired [1-3]. In our series the average age of our patients is 28 years. We recall that the fineness of the bone walls of the frontal sinus which separate it from the anterior cerebral fossa on the one hand, and the orbital structures on the other hand, as well as the possible presence of anatomical variants to the type of spontaneous dehiscence of a Sinusian lining will facilitate the propagation of the infection towards the endocranium and / or the orbit by contiguity (step by step through osteitis or bone dehiscence) or by continuity (venous vascular pathway) [1, 2]. Imaging provides considerable help in the accurate diagnosis of these complicated sinusites and in the analysis of favorable anatomical factors. In our study, the fracture line of the external table of the frontal sinus, caused by an old craniofacial trauma, was well demonstrated in two patients who had a frontal sinusitis complicated with frontal osteomyelitis. In a study of 24 children with complications of acute sinusitis, the scannographic data on length and infundibular width were compared with those of 196 uninfected children. The authors deduced that in children with sinus complications the infundibular length was shorter and its width was greater than in healthy patients [8].

Radiological aspects characteristic of the main complications of acute frontal sinusitis [5, 6, 9]: the main complications that can occur during acute bacterial frontal sinusitis are: frontal osteomyelitis; endocranial complications (empyema, abscess, thrombophlebitis, meningitis); oculo-orbital complications; and cellulites infiltrating the soft tissues of the frontal site. These different complications can be associated in the same patient. In our series 40% of patients had more than one complication. This is the example of the patient who had a periosteal frontal abscess associated with an extradural empyema Figure 5. Another patient had a right frontal-parietal extradural empyema associated with a thrombophlebitis of the upper longitudinal sinus Figure 6. The incidence of each of these complications in frontal sinusitis is not well known. However, after a review of the literature, it appears that intracranial complications are the most frequent, followed by frontal osteomyelitis, followed by orbital complications [1, 4-6, 9]. The Computed Tomography of the facial and cerebral mass, with injection of contrast product, is the reference examination, to order of first intention to explore these extra-sinusian infectious extensions. It must be urgently carried out on the slightest clinical suspicion of complicated sinusitis [1, 4, 6]. The technique must be rigorous: thin axial cuts, high resolution, multi-planar reconstruction, double fenestration (bone + parenchymatous) and enlarged window in search of venous complications. MRI is recommended for suspected endocranial involvement [1, 6, 10-12] and should be performed according to a well-defined protocol: for endocranial exploration, T1-weighted sequences after Gadolinium injection, Diffusion Weighted Imaging (DWI) sequences and FLAIR (Fluid Attenuated Inversion Recovery) sequences. DWI sequences are known to be highly sensitive to detecting foci of encephalic suppuration and FLAIR in the detection of edema and meningitis [4, 13, 14]. So far, no comparative study has been carried out to evaluate the diagnostic reliability of CT compared to MRI in terms of complicated sinusitis [4]. Ch. S. Betz et al [6] concluded after their study of 12 patients who had complicated frontal sinusitis that the CT of the facial mass injected is the basic examination which makes it easy to make the diagnosis of the complication of a frontal sinusitis, and MRI appears to become mandatory on suspicion of intracranial extension. This deduction is reinforced by the advantage of CT by its speed of acquisition without the need for sedation (especially in children) compared to the MRI which imposes sedation with a longer examination time, while taking into account its high cost, and its lack of accessibility in an emergency context. The endocranial infectious extension from the frontal sinus can be directed to the anterior cerebral fossa directly by bone dehiscence or osteomyelitis of the frontal bone, which may give subdural or extradural empyema, intracerebral abscess -parenchymal cells often located in the frontal lobe [6], meningitis, cerebral thrombophlebitis or even cranial pairs. The subdural and extradural empyema represent two extra-axial suppurative collections, located between the cranium and the cerebral parenchyma, which present to the imagery a characteristic peripheral enhancement. They can sometimes cause a mass effect on adjacent brain tissue with an edematous reaction [4, 14]. The differentiation between extradural and subdural empyema can be difficult. Indeed, on the CT, if the empyema crosses in front the falsity of the brain, this indicates that the empyema is of extradural localization. And if it persists unilaterally in relation to the falsity of the brain or extends posteriorly along, it is rather of subdural site [4]. These empyemes show little diffusion on DWI sequences at MRI [4] because of the high concentration of purulent collections in altered polynuclear cells and the very thick character of their contents.

If the infection still extends from the extra-axial endocranial space to the intra-parenchymatous one, foci of encephalitis and then of cerebral abscesses can be formed. At an early stage, pre-suppurative encephalic lesions are difficult to detect by CT, which can only mount a hypodense focus with a mass effect and sometimes some scattered enhancement. MRI is more sensitive to detect these pre-suppurative lesions not detected on the CT scan due to its T2 and FLAIR-weighted sequences, which show them in hypersignal [4, 14]. Intracranial abscesses, such as empyema, have poor diffusion on DWI sequences. MRI is also more sensitive to reveal localized thickening and localized meningeal enhancement (pachymeningitis). In its FLAIR images, a characteristic linear hypersignal can be visualized in relation to a thickening of the meninges [4]. A more precise analysis of the cavernous sinus is obtained by angio-MRI [15]. In rare cases, the osteomyelitis of the frontal bone can be externally presented in the form of a subperiosteal abscess, which manifests itself clinically by infiltration and "pseudotumoral" bulging of the soft tissues in the anterior frontal region, Called Pott's tumor or Pott's Puffy Tumor [4, 6, 12, 16]. This entity was described for the first time in the 18th century by "Percivall Pott", and was labelled as a serious complication of frontal sinusitis given the potentially fatal endocranial extension risk [12]. On the CT, one can visualize the periosteal collection in the form of a hypodense image with peripheral enhancement facing the anterior wall of the frontal sinus, and the communication between the latter and the facing collection is often identifiable on the CT scan of lytic hypodense zone characteristic of bone destruction [4]. A case of Pott's Puffy Tumor has been identified in our series. Orbital complications of frontal sinusitis, like other forms of complicated sinusitis, join the Chandler classification which subdivides them into 5 categories [17]. CT with Contrast Injection, due to its speed of access, is the examination of choice to explore these orbital infections, to specify their precise site pre-septal or retro-septal [1] and to allow to choose the initially surgical pathway when drainage is indicated [1, 4, 18]. If the tomodensitometric data were not obvious from orbital cellulitis, MRI may be useful by its T2-weighted sequence with fat saturation (T2 FAT SAT) which is highly sensitive to the detection of orbital inflammatory processes. DWI diffusion sequences, in addition, make it possible to differentiate orbital cellulitis from other radiologically overlapping pathologies such as orbital inflammatory syndrome and lymphomatous lesions [4, 19]. When periosteal and intra-orbital abscesses are both on the MRI in isosignal T1, hypersignal T2 with peripheral enhancement and low diffusion on DWI [4, 20]. Thrombosis of the cavernous sinus is considered both an orbital and intracranial complication [4, 21]. Its positive diagnosis can be made by CT or MRI showing a lacunar image in the cavernous sinus and / or a convexity of its walls which translates a mass effect. A lacunar image or dilation of the superior ophthalmic vein is also an important factor in favor of cavernous sinus thrombosis [4]. In summary, the diagnosis of suppurative complications of acute frontal sinusitis is greatly aided by imaging data based on CT, performed systematically in all cases, sometimes supplemented by MRI when intracranial complication is suspected for better parenchymatous cerebral and meningeal analyses. The imaging allows, in addition, to choose the approach first when a surgical drainage is indicated and to monitor the subsequent evolution.

## Conclusion

The complications of frontal bacterial sinusitis are severe, threatening the functional and even vital prognosis. Cross-sectional imaging, dominated by CT with injection, is the fundamental tool in the diagnosis of complicated frontal sinusitis. MRI is indicated on suspicion of intracranial complication for better cerebral and meningeal parenchymal analysis. Imaging helps in choosing the surgical approach when surgical drainage is indicated and in subsequent monitoring. We insist on urgent and multidisciplinary care involving ENT surgeons, radiologists, neurosurgeons, ophthalmologists, and infectiologists.

### What is known about this topic

- Even though being a rare condition in the era of antibiotic treatment, complications of acute frontal sinusitis still pose a potentially life-threatening problem;
- Cross-sectional imaging is important in early and accurate diagnosis of complicated frontal sinusitis;
- A close cooperation with the related specialties: ENT, radiology, neurosurgery, ophthalmology is necessary.

### What this study adds

- The aim of this publication is to deliver up to date information on modern diagnostic options of these complications;
- Our results demonstrate that the most common complications of frontal sinusitis was the Frontal osteomyelitis which is in accordance with the findings of the previous studies;
- We noted that 40% of our patients had two types of associated complications.
